# Ultra-low-frequency wave-driven diffusion of radiation belt relativistic electrons

**DOI:** 10.1038/ncomms10096

**Published:** 2015-12-22

**Authors:** Zhenpeng Su, Hui Zhu, Fuliang Xiao, Q.-G. Zong, X.-Z. Zhou, Huinan Zheng, Yuming Wang, Shui Wang, Y.-X. Hao, Zhonglei Gao, Zhaoguo He, D. N. Baker, H. E. Spence, G. D. Reeves, J. B. Blake, J. R. Wygant

**Affiliations:** 1 CAS Key Laboratory of Geospace Environment, Department of Geophysics and Planetary Sciences, University of Science and Technology of China, Hefei, Anhui 230026, China; 2 Collaborative Innovation Center of Astronautical Science and Technology, University of Science and Technology of China, Hefei, Anhui 230026, China; 3 Mengcheng National Geophysical Observatory, School of Earth and Space Sciences, University of Science and Technology of China, Hefei, Anhui 230026, China; 4 School of Physics and Electronic Sciences, Changsha University of Science and Technology, Changsha Hunan 410004, China; 5 Institute of Space Physics and Applied Technology, Peking University, Beijing 100871, China; 6 Synergetic Innovation Center of Quantum Information and Quantum Physics, University of Science and Technology of China, Hefei, Anhui 230026, China; 7 Harbin Institute of Technology Shenzhen Graduate School, Shenzhen, Guangdong 518055, China; 8 Laboratory for Atmospheric and Space Physics, University of Colorado Boulder, Boulder, Colorado 80303-7814, USA; 9 Institute for the Study of Earth, Oceans, and Space, University of New Hampshire, Durham, New Hampshire 03824-3525, USA; 10 Space Science and Applications Group, Los Alamos National Laboratory, Los Alamos, New Mexico 87544, USA; 11 The Aerospace Corporation, Los Angeles, California 90245-4609, USA; 12 School of Physics and Astronomy, University of Minnesota, Minneapolis, Minnesota 55455, USA

## Abstract

Van Allen radiation belts are typically two zones of energetic particles encircling the Earth separated by the slot region. How the outer radiation belt electrons are accelerated to relativistic energies remains an unanswered question. Recent studies have presented compelling evidence for the local acceleration by very-low-frequency (VLF) chorus waves. However, there has been a competing theory to the local acceleration, radial diffusion by ultra-low-frequency (ULF) waves, whose importance has not yet been determined definitively. Here we report a unique radiation belt event with intense ULF waves but no detectable VLF chorus waves. Our results demonstrate that the ULF waves moved the inner edge of the outer radiation belt earthward 0.3 Earth radii and enhanced the relativistic electron fluxes by up to one order of magnitude near the slot region within about 10 h, providing strong evidence for the radial diffusion of radiation belt relativistic electrons.

## Abstract (web summary)

The Van Allen radiation belts are two zones of energetic particles encircling the Earth, but how electrons are accelerated to relativistic energies remains unclear. Here, the authors analyse a radiation belt event and provide evidence in favour of the ULF wave-driven radial diffusion mechanism.

## Graphical abstract

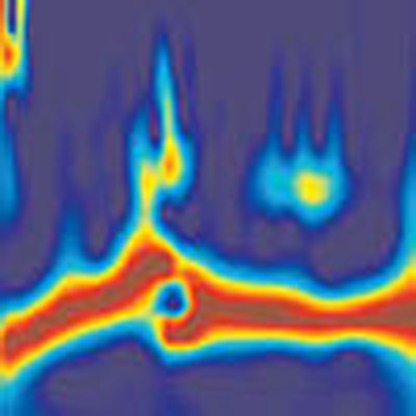

The geomagnetic field geometry allows three quasi-periodic motions of outer Van Allen radiation belt^1^ relativistic electrons over distinct timescales: gyration about the magnetic field line on a timescale of milliseconds; bounce along the magnetic field line between two magnetic mirror points on a timescale of seconds; drift circling the Earth on a timescale of kiloseconds. Each periodicity gives rise to an approximate constant of motion defined as the adiabatic invariant. Surrounding the highly dynamic outer radiation belt^2^, one outstanding question has been how electrons are accelerated to relativistic energies of several million electron volts. Two invariant-violating processes have been proposed: local acceleration by VLF (∼kHz) chorus waves^3^,^4^ and radial diffusion by ULF (∼mHz) waves^5^,^6^,^7^,^8^,^9^. On 30 August 2012, National Aeronautics and Space Administration launched a twin-spacecraft mission, Van Allen Probes (formerly known as the Radiation Belt Storm Probes (RBSP))^10^, to make the measurements for the identification of acceleration mechanisms. Recent analyses^11^,^12^ of the Van Allen Probes data have presented compelling evidence for the local acceleration in the heart of the outer radiation belt. However, the importance of radial diffusion for the radiation belt evolution has not yet been determined definitively.

ULF waves have been thought to effectively violate the third adiabatic invariant L* under the drift-resonance condition^5^ *ω*=*mω*_d_ (with the wave frequency *ω*, the azimuthal wave mode number *m* and the electron drift frequency *ω*_d_). The quantity L* is the Roederer's drift-shell parameter^13^, equal to the equatorial radial distance (made dimensionless by Earth radii *R*_E_) of the adiabatically equivalent electron drift orbit in the dipole field. Violation of the third invariant (but conservation of the first two invariants) causes the radial migration of resonant electrons and consequently the variation of their energies and pitch angles. The electrons in drift resonance with broadband ULF waves move stochastically along the radial direction, which is described by the radial diffusion theory^5^,^7^,^8^. This theory was indirectly supported by the strong correlation between ULF wave power and radiation belt electron flux enhancement^14^,^15^,^16^,^17^,^18^, but due to the limitations in previous particle/field observations, the *in situ* wave–particle interaction characteristics were lacking to clarify the associated physical process. Particularly, previous works^7^,^14^,^15^,^16^,^19^,^20^ often concentrated on the radiation belt reformation events during the geomagnetic storms. These geomagnetic storms did cause marked dynamics of energetic electrons, but the strongly disturbed magnetosphere very likely allowed the concurrence^20^,^21^,^22^ of ULF wave-driven radial diffusion and VLF chorus wave-driven local acceleration. The superposition of two processes is not conducive to isolating the contribution of radial diffusion.

Here we report a unique radiation belt event serendipitously observed by the Van Allen Probes in the plasmasphere during non-storm times. The plasmasphere is a torus-shaped region of cold and dense plasma surrounding the Earth, where the VLF chorus wave-driven local acceleration seldom occurs. By analysing the high-resolution data and performing the detailed simulation, we demonstrate that the radial diffusion by intense ULF waves was responsible for the radiation belt relativistic electron evolution in this event.

## Results

### Wave modulation of relativistic electrons

**Figure 1:**
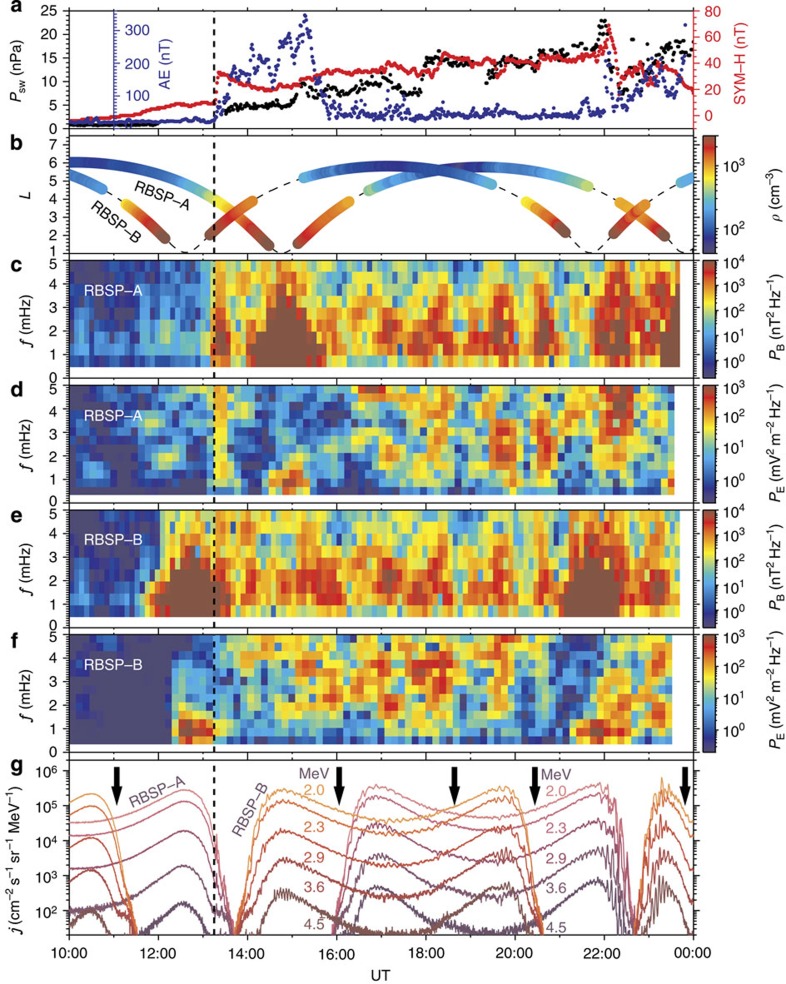
An overview of the 15 February 2014 radiation belt event. (**a**) Solar wind dynamic pressure *P*_sw_, geomagnetic activity indices AE (measuring the substorm intensity) and SYM-H (measuring the storm intensity and partially reflecting the variation of solar wind dynamic pressure). (**b**) Cold electron number density *ρ* from the EFW instrument. The density were always beyond 40 cm^−3^, indicating the locations of Van Allen Probes in the plasmasphere. (**c**,**e**) Power spectral density *P*_B_ of the compressional ULF wave magnetic field in the MFA coordinate system from the EMFISIS magnetometer. (**d**,**f**) Power spectral density *P*_E_ of the *y* component ULF wave electric field in the mGSE coordinate system from the EFW instrument. In the outer belt, the ULF wave power were intensified obviously by the solar wind variation after 13:15 UT (vertical-dashed line). (**g**) Spin-averaged differential electron fluxes *j* (colour-coded according to energy) in the outer radiation belt from the REPT instrument. Black arrows mark the times around which the regular oscillations of fluxes were relatively weak.

**Figure 2:**
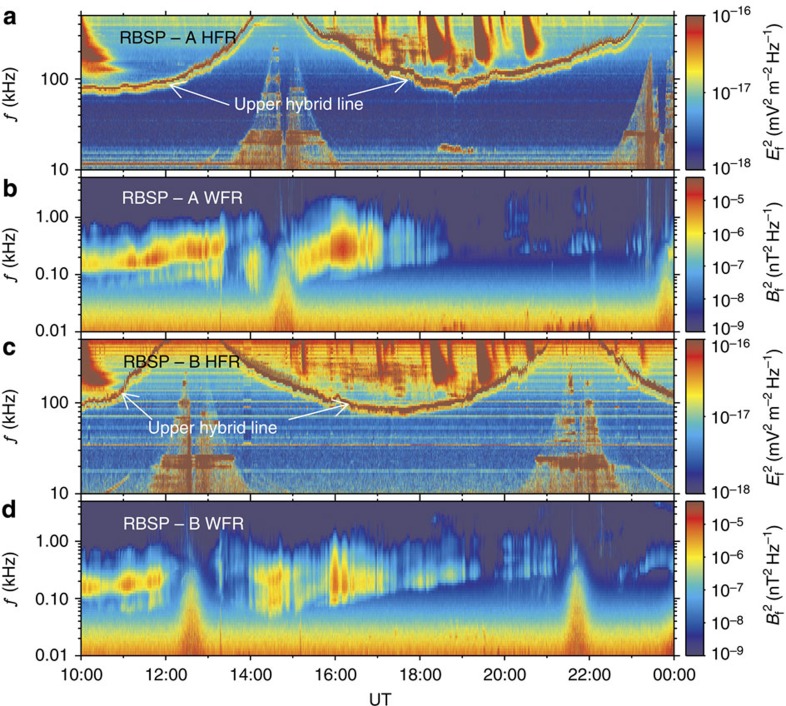
Electromagnetic power spectral densities of high-frequency and very-low-frequency waves. (**a**,**c**) Wave electric power spectral density from the high-frequency receiver (HFR) of the EMFISIS Suite and Integrated Science suite. The upper hybrid resonance bands (bright lines) had frequencies (positively correlated with the background electron density^59^) beyond 70 kHz, indicating that the twin spacecrafts stayed in the high-density plasmasphere for the entire orbits. (**b**,**d**) Wave magnetic power spectral density from the Waveform Receiver (WFR) of the EMFISIS suite. There were VLF hiss waves in the frequency range 0.1–1.0 kHz contributing to the slow loss of relativistic electrons, but no VLF chorus waves responsible for the local acceleration of relativistic electrons.

The radiation belt event of interest occurred on 15 February 2014, with the required particle/field data collected by the Relativistic Electron–Proton Telescope (REPT)^23^ and the Magnetic Electron Ion Spectrometer (MagEIS)^24^ of the Energetic particle, Composition and Thermal plasma (ECT) instrument suite^25^, the Electric Fields and Waves (EFW) instruments^26^ and the Electric and Magnetic Field Instrument Suite and Integrated Science (EMFISIS) instrument suite^27^ on board the Van Allen Probes. During the event, the magnetosphere was free from magnetic storms but experienced some weak and short-duration substorms (Fig. 1a). Such magnetospheric conditions favoured the expansion of plasmasphere to at least *L*=6.5 (Figs 1b and 2a,c; Supplementary Table 1). The quantity *L* is the McIlwain's drift-shell parameter^28^, equal to the equatorial radial distance (made dimensionless by Earth radii *R*_E_) of the drift orbit of the electron having the same mirror field, second invariant and energy in the dipole field. In the plasmasphere, there were no VLF chorus waves contributing to the local acceleration (Fig. 2b,d). In the time range from 13:15 to 24:00 UT, the solar wind dynamic pressure (Fig. 1a) exhibited continuous fluctuations especially around 13:15 and 22:00 UT. The solar wind variation drove the generation of ULF waves over a wide frequency range in the outer radiation belt (Fig. 1c–f). Correspondingly, the relativistic (2.0–4.5 MeV) electron fluxes oscillated periodically (Fig. 1g), which is one of the expected drift-resonance characteristics^29^. In contrast to the previously reported ULF modulation^30^,^31^ of electron fluxes at the relatively low energies and/or in a localized spatiotemporal region, this event presented the ULF modulation of highly relativistic electron fluxes for about 10 h throughout the outer radiation belt, serving as an observational evidence for the global and long-lasting drift resonance between ULF waves and relativistic electrons.

**Figure 3:**
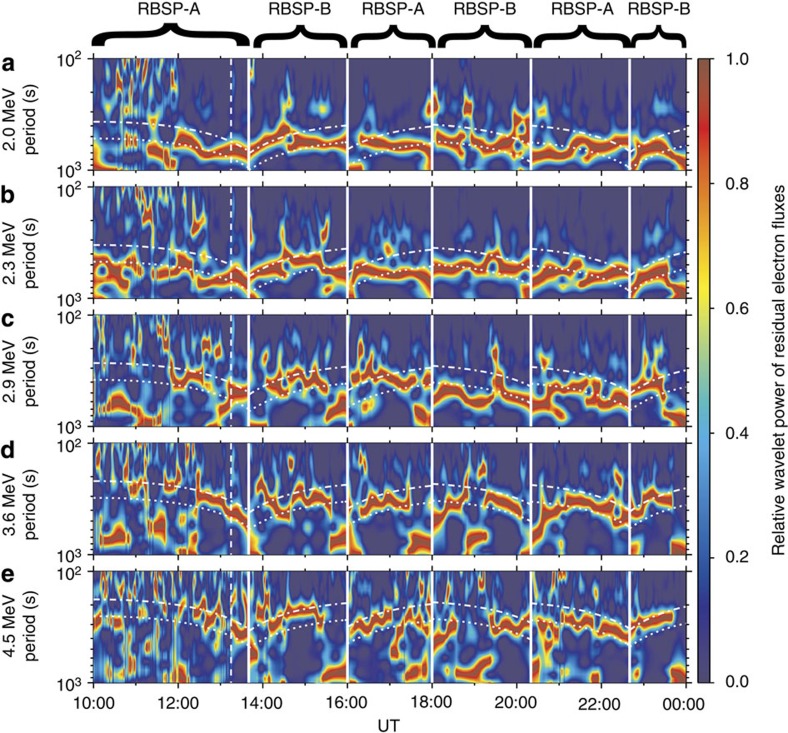
Relative wavelet power of residual electron fluxes. (**a**-**e**) Relative wavelet power at the energy channels 2.0, 2.3, 2.9, 3.6 and 4.5 MeV, respectively. Residual flux is defined as (*j*−*j*_0_)/*j*_0_ with *j* the spin-averaged differential flux from the REPT instrument and *j*_0_ the 1,000 s running averaged *j*, which reflects the oscillation of electron flux at each energy channel. Relative wavelet power is defined as the wavelet power of residual flux normalized by the maximum power with oscillation period <1,000 s. The obtained relative wavelet power (colour-coded scale) of the twin spacecrafts is plotted alternatively to characterize the oscillation of the outer radiation belt electron fluxes. The superposed dotted and dot-dashed lines represent the drift periods of electrons with the equatorial pitch angles 0° and 90° at the corresponding energy channels in the dipole field. The vertical-dashed line denotes the beginning of ULF wave enhancement induced by the solar wind dynamic pressure variation.

The azimuthal mode number *m* of ULF waves is required to quantify the drift-resonance process. Wavelet transforms^32^ have been performed on the electron fluxes at different energy channels to identify their dominant oscillation periods (Fig. 3). During some time intervals (10:00–13:15, 15:30–16:00, 17:30–19:30, 20:20–21:00 and 23:30–24:00 UT), the regular oscillations of fluxes were so weak (as marked by the black arrows in Fig. 1g) that the wavelet transforms mainly captured the power associated with the irregular noise and/or the radial variation of fluxes. Most of the time the dominant oscillation periods of electron fluxes are found to change with energy and location but remain close to the corresponding electron drift periods, which can be considered as the result of the *m*=1 mode drift-resonance between relativistic electrons and broadband ULF waves^29^,^33^. It should be mentioned that recent magnetohydrodynamic simulation^34^ for a magnetic storm had suggested the dominance of *m*=1 mode ULF waves in the radiation belt region.

### Global evolution of relativistic electrons

**Figure 4:**
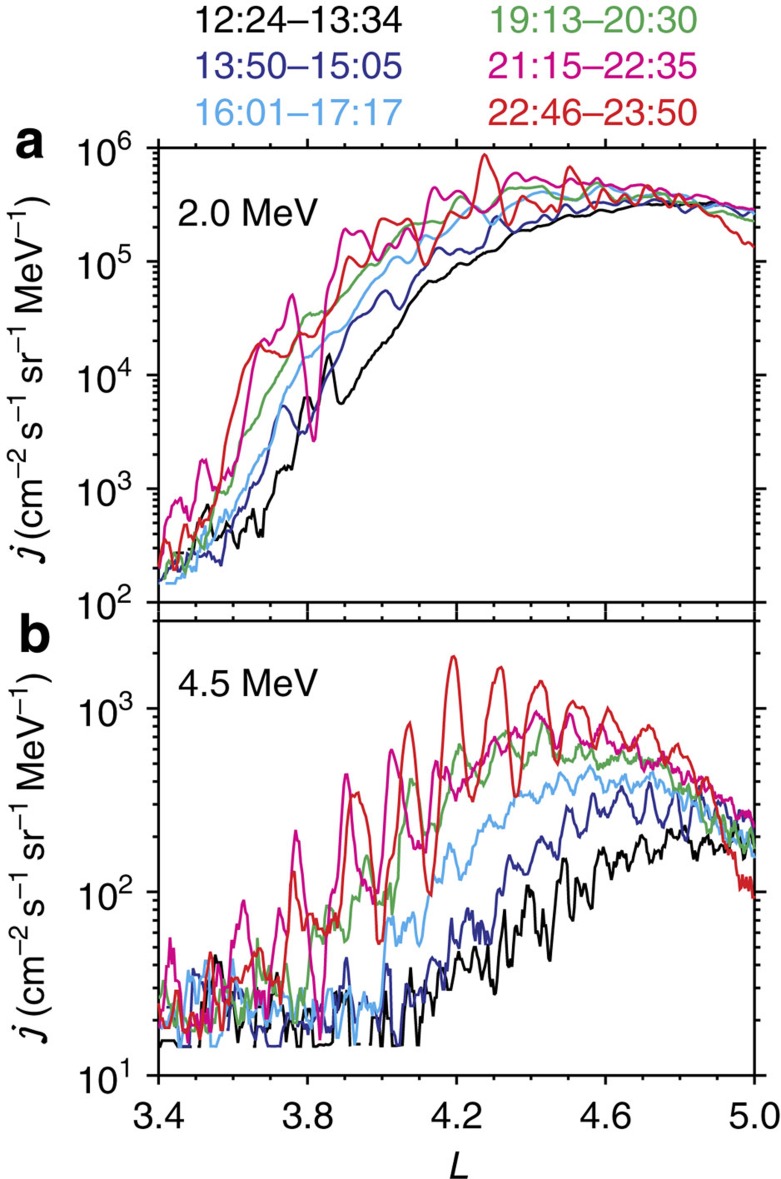
Radial profiles of relativistic electron fluxes for six outer radiation belt passages. Each colour-coded profile shows the *L*-dependent differential flux *j* from REPT instrument during a passage. (**a**,**b**) Differential fluxes of electrons with the equatorial pitch angles 55° at the energy channels 2.0 and 4.5 MeV, respectively. Because of the latitudinal variation of twin spacecrafts, the corresponding local pitch angles varied between 55° and 90° in the TS04D geomagnetic field model^60^. Electron fluxes at the other energy channels 2.3, 2.9 and 3.6 MeV exhibited the similar characteristics.

**Figure 5:**
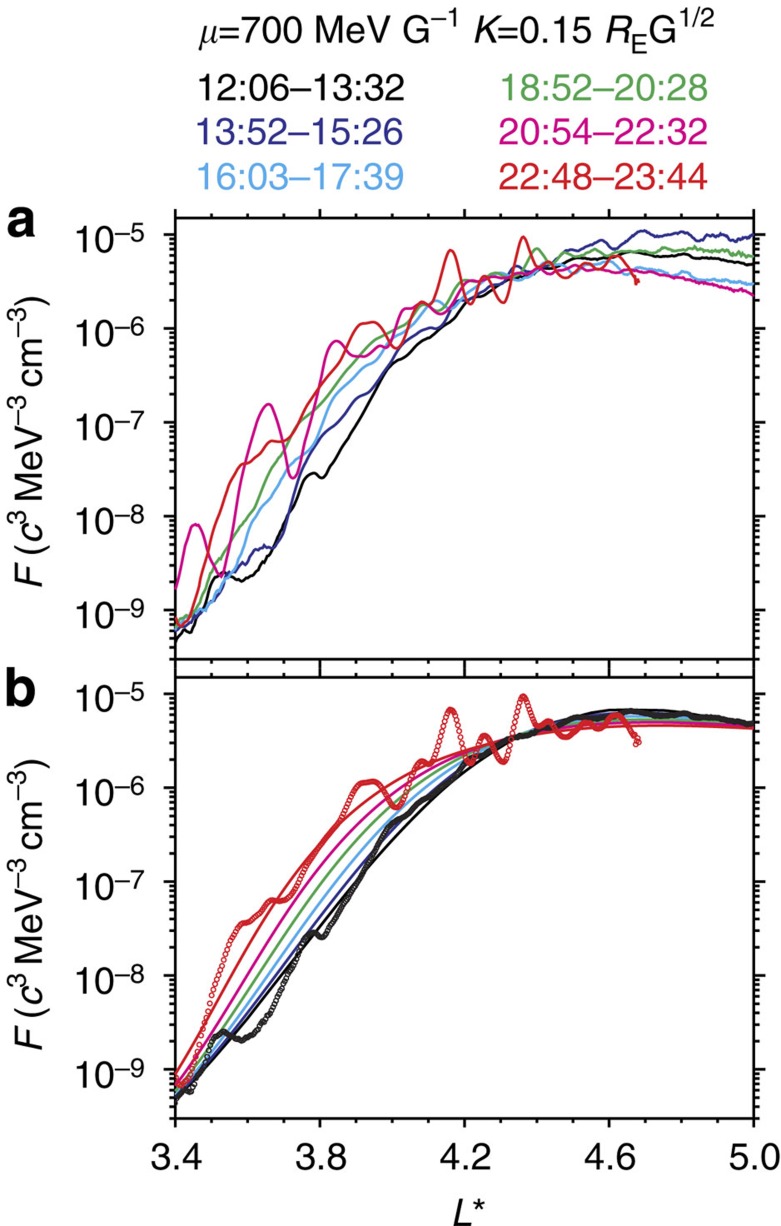
Radial profiles of relativistic electron phase space densities for six outer radiation belt passages. Each colour-coded profile shows the L*-dependent phase space density *F* at the fixed first adiabatic invariant *μ*=700 MeV G^−1^ and second adiabatic invariant *K*=0.15 *R*_E_G^1/2^ during a passage. The corresponding energies were about 1.0 MeV at L*=5.5 and 2.5 MeV at L*=3.4, and the corresponding local pitch angles were 40°–70°. (**a**) Observations (lines) from MagEIS and REPT instruments in the TS04D geomagnetic field model. (**b**) Simulations (lines) from the radial diffusion equation, overplotted with the observations (circles) for ease of comparison.

**Figure 6:**
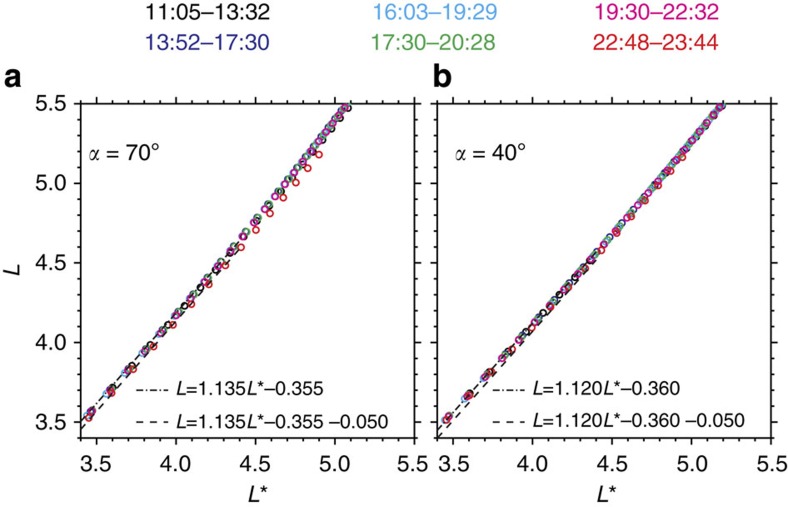
Mapping relations between *L* and L* for six outer radiation belt passages. Mapping relations (circles colour-coded according to the time) are calculated every 300 s in the TS04D geomagnetic field model. (**a**,**b**) Mapping relations at two different local pitch angles 70° and 40°, respectively. The dot-dashed and dashed lines are overplotted to identify the variation of the mapped *L* with time for the fixed L* near the slot region. Similar characteristics can be found in the mapping relations at the other local pitch angles.

The Van Allen Probes passed through the radiation belt six times in the time range of interest. The comparison of the *L*-dependent electron fluxes observed during each passage is made to identify the global evolution of radiation belt electrons (Fig. 4). Within about 10 h, the inner edge of the outer belt moved inward about 0.3 *R*_E_ at 2.0 MeV and 0.8 *R*_E_ at 4.5 MeV. Around the inner edge, the electron fluxes increased by an average factor of up to 10. To exclude the possible adiabatic effect associated with the compression of magnetosphere, these electron fluxes are transformed into phase space density (PSD) in the adiabatic invariant coordinate system (Fig. 5a; Supplementary Figs 1–3; Supplementary Note 1). For the fixed L* near the slot region, the variation of mapped *L* with time was below 0.05 throughout the event (Fig. 6). The initial PSD peaked at the large L* (that is, the external source region) and monotonically decreased with the decreasing L*. As time went on, the external source showed some fluctuations. At the centre of the outer belt (L*≈4.3), the PSD remained unchanged, once again demonstrating the absence of local acceleration. In the slot region, the inner edge of the outer belt was transported inward about 0.3 *R*_E_. Specifically, at L*≈3.6, the PSD gradually increased by one order of magnitude within 10 h. These global evolution characteristics were qualitatively consistent with the prediction of radial diffusion theory^35^,^36^. It should be mentioned that the very strong fluctuations of electron PSDs after 22:00 UT were perhaps associated with the coherent transport process^33^.

**Figure 7:**
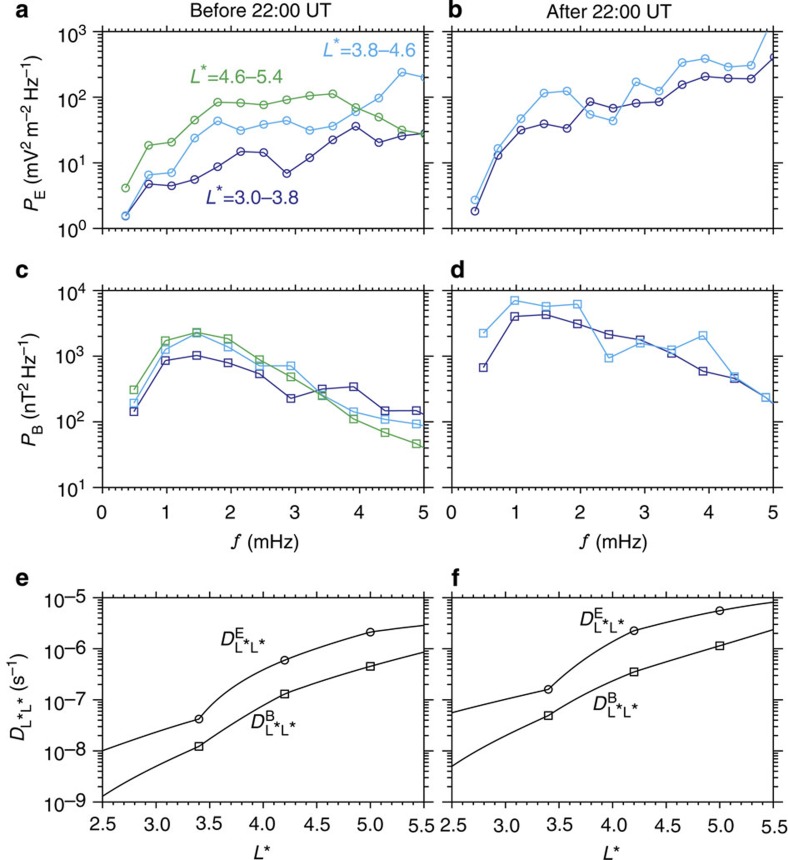
Electromagnetic power spectral densities of ultra-low-frequency waves and radial diffusion rates. (**a**–**d**) Averaged electric *P*_E_ (circles) and magnetic *P*_B_ (squares) power spectral densities in three different regions before and after 22:00 UT. Solid lines are introduced to guide the eye. (**e**,**f**) Radial diffusion rates (lines) from the electric 
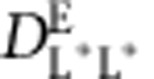
 and magnetic 
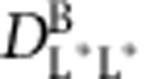
 perturbations. Symbols are drawn to mark the diffusion rates at the centres of the three spatial regions. The total radial diffusion rates 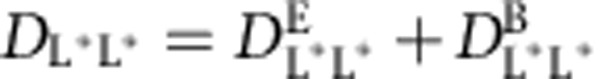 were the sum of the electric and magnetic radial diffusion rates.

The radial diffusion of radiation belt electrons is simulated by solving the equation^37^


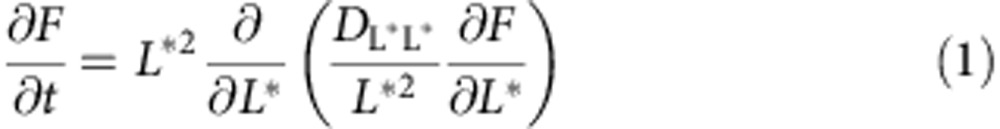


with the electron PSD *F* and the radial diffusion rate *D*_L*L*_ calculated from the observed ULF waves^9^ (Fig. 7). The simulations (Fig. 5b; Supplementary Fig. 4; Supplementary Note 2) well reproduce the average rate and extent of the observed PSD variations. The radial diffusion process can effectively reduce the electron PSD gradients^35^. In the region L*>4.3, the electron PSD behaved smoothly and consequently presented minor changes. In contrast, the electron PSD possessed a very steep gradient in the region L*<4.3 and then allowed the significant enhancement.

## Discussion

There was an average enhancement of relativistic electron fluxes by up to one order of magnitude within about 10 h on 15 February 2014, which could be easily misinterpreted in the framework of the VLF chorus wave-driven local acceleration. The high-resolution data and the detailed simulation clearly show that, in the absence of VLF chorus waves, the ULF waves could radially diffuse and effectively accelerate the radiation belt electrons in this event. The ULF waves^38^,^39^,^40^,^41^,^42^,^43^,^44^, as well as the VLF chorus waves^45^,^46^,^47^,^48^,^49^,^50^,^51^,^52^,^53^,^54^, are commonly observed in the magnetosphere. To provide a more comprehensive picture, we additionally analyse two more radiation belt events with the concurrence of ULF and VLF chorus waves (Supplementary Figs 5–14; Supplementary Tables 2 and 3; Supplementary Note 3). During the 18 January 2013 event, there were moderate ULF waves but quite weak VLF chorus waves. The radial diffusion, dominating over the local acceleration, caused the earthward movement of the inner edge of the outer radiation belt. In contrast, both moderate ULF waves and strong VLF chorus waves occurred during the 22 September 2014 event. The local acceleration produced the relativistic electron PSD peaks, and the radial diffusion redistributed the electrons along the radial direction to reduce the PSD gradients.

Our results present evidence for the importance of ULF wave-driven radial diffusion in the outer radiation belt dynamics. Under differing magnetospheric conditions, the accurate contributions of local acceleration and radial diffusion to the radiation belt evolution should be carefully examined. The radial diffusion can play a dominant role in some radiation belt electron acceleration events (such as the one discussed here). Compared with the local acceleration in the previously reported marked events^11^,^12^,^21^, the radial diffusion in the present event yielded a smaller acceleration rate and a smaller relativistic electron flux enhancement. However, no matter how strong the action of local acceleration is, the additional redistribution of relativistic electrons by the radial diffusion would constitute an important part of the outer radiation belt dynamics^11^,^33^,^35^,^55^,^56^,^57^.

## Methods

### Calculation of wave power spectral density

We use the level-3 magnetic field data with the time resolution of 1 s in the Geocentric Solar Magnetospheric coordinate system to obtain the magnetic power spectral density of ULF waves. A three-step procedure is performed as follows. First, the magnetic field data are running averaged over 11 s to reduce the spin modulation of Van Allen Probes^44^. Then, these magnetic field data are projected on the so-called mean field aligned (MFA) coordinates^58^. In the MFA coordinate system, the parallel direction is determined by the 2,048-s running average of the instantaneous magnetic field, the azimuthal direction is obtained by the cross product of the parallel vector and the satellite position vector, and the radial direction completes the triad. This system is used to separate magnetic field perturbations into the toroidal (azimuthal), poloidal (radial) and compressional (parallel) components^26^. Finally, an overlapped (80%) 2,048-point fast Fourier transform is adopted to calculate the power spectral densities of each magnetic field components. The obtained power spectra densities have the time resolution of 6.83 min and the frequency resolution of 0.49 mHz. We use the level-3 electric field data with the time resolution of 10.9 s in the modified geocentric solar ecliptic (mGSE) coordinate system to obtain the electric power spectral density of ULF waves. The mGSE unit vectors (**X**_mGSE_, **Y**_mGSE_ and **Z**_mGSE_) can be generally expressed in terms of the GSE unit vectors (**X**_**GSE**_, **Y**_**GSE**_ and **Z**_**GSE**_) and the spin axis unit vector **S**_GSE_: **X**_mGSE_=**S**_GSE_, 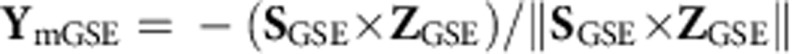, and **Z**_mGSE_=**X**_mGSE_ × **Y**_mGSE_. When the spin axis points towards the sun (**S**_GSE_=**X**_GSE_), the mGSE system is exactly the same as the GSE system. The EFW instrument only measured the two electric field components in the spin plane, and the third component has to be derived from the assumption **E**·**B**=0. For this event after 22:00 UT, the magnetic field component *B*_*x*_ of RBSP-B in the mGSE system was nearly zero and the electric field component *E*_*x*_ was unconstrained by the **E**·**B**=0 condition, not allowing the calculation of electric field vectors in the MFA system. Note that the most significant ULF waves happened to be generated after 22:00 UT. Hence, we calculate the power spectral densities of the electric field components in the mGSE system (rather than in the MFA system) to ensure the continuous monitoring of ULF waves throughout this event. Specifically, an overlapped (80%) 256-point fast Fourier transform is performed on the electric field data (with the preliminary substraction of the co-rotation electric field and the **V**_sc_ × **B** electric field induced by the satellite motion through the Earth's magnetic field), and the obtained the electric field power spectral densities possess the time resolution of 9.3 min and the frequency resolution of 0.36 mHz. It should be mentioned the power spectral densities exhibited unphysical intensification^44^ associated with the steep variation of the background magnetic field during the averaging time of 2,048 s in the region *L*<3 or the contamination by the large **V**_sc_** × B** electric field in the region *L*<2.5.

### Calculation of radial diffusion rates

The radial diffusion is generally produced by both the ULF electric and magnetic perturbations. In this event, the *m*=1 mode symmetric drift resonance is suggested to be dominant. The corresponding radial diffusion rates are written as^9^


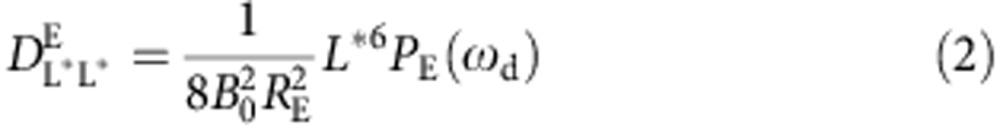



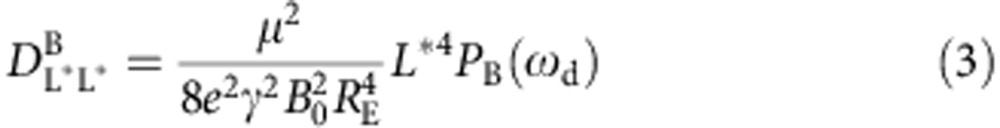


with the equatorial magnetic field magnitude at the Earth's surface *B*_0_=0.312 G, the Lorentz factor of electrons *γ*, the Earth's radii *R*_E_, and the power spectral densities of the azimuthal wave electric field *P*_E_ and the compressional wave magnetic field *P*_B_ at the resonant frequency *ω*=*ω*_d_ in the MFA system. As discussed in the previous section, the azimuthal wave electric field cannot be obtained continuously. In this study, *P*_E_ in the MFA system is approximated as the power spectral density of the *y* component electric field in the mGSE system. We calculate the radial diffusion rates in two time ranges (14:00–22:00 and 22:00–24:00 UT). In each time range, we average the wave power in three spatial regions (L*=3.4±0.4, 4.2±0.4 and 5.0±0.4) and then obtain the L*-dependent ULF wave power distribution through the linear interpolation. In the inner region L*<3.4, the wave power is assumed to equal that at L*=3.4; in the outer region L*>5.0 before 22:00 UT or L*>4.2 after 22:00 UT, the wave power is assumed to equal that at L*=5.0 or 4.2. Note that there were no ULF wave data in the region L*=5.0±0.4 after 22:00 UT. These assumptions would not significantly affect our conclusions, since the ULF wave-driven variation in electron PSDs mainly occurred in the region L*=3.4–4.2. In the frequency range 1–5 mHz, the obtained electromagnetic power spectral densities (Fig. 7a–d) were comparable to the previous statistical results^38^,^39^,^40^,^41^,^43^. The obtained electric diffusion rates dominated over the magnetic diffusion rates (Fig. 7e,f), consistent with the previous calculations^38^,^40^,^43^.

### Simulation of radiation belt evolution

The simulation code is extracted from our previously developed STEERB model^37^. The fully implicit finite difference method is adopted to solve the radial diffusion equation with the computational domain 2.5≤L*≤5.5. The initial condition is given by the observed electron PSD during the first passage (12:06–13:32 UT) of the outer radiation belt. The electron PSD is assumed to be fixed at the outer boundary L*=5.5 and to be zero at the inner boundary L*=2.5.

### Software availability

The software for wavelet analysis is available at http://paos.colorado.edu/research/wavelets/.

## Additional information

**How to cite this article:** Su, Z. *et al.* Ultra-low-frequency wave-driven diffusion of radiation belt relativistic electrons. *Nat. Commun.* 6:10096 doi: 10.1038/ncomms10096 (2015).

## Supplementary Material

Supplementary Information Supplementary Figures 1-14, Supplementary Tables 1-3, Supplementary Notes 1-3 and Supplementary References

## References

[b1] Van Allen J. A. & Frank L. A. Radiation around the Earth to a radial distance of 107,400 km. Nature 183, 430–434 (1959).

[b2] Friedel R. H. W., Reeves G. D. & Obara T. Relativistic electron dynamics in the inner magnetosphere - a review. J. Atmos. Sol. Terr. Phys 64, 265–282 (2002).

[b3] Horne R. B. & Thorne R. M. Potential waves for relativistic electron scattering and stochastic acceleration during magnetic storms. Geophys. Res. Lett. 25, 3011–3014 (1998).

[b4] Summers D., Thorne R. M. & Xiao F. Relativistic theory of wave-particle resonant diffusion with application to electron acceleration in the magnetosphere. J. Geophys. Res. 103, 20487 (1998).

[b5] Fälthammar C.-G. Effects of time-dependent electric fields on geomagnetically trapped radiation. J. Geophys. Res. 70, 2503–2516 (1965).

[b6] Elkington S. R., Hudson M. K. & Chan A. A. Acceleration of relativistic electrons via drift-resonant interaction with toroidal-mode Pc-5 ULF oscillations. Geophys. Res. Lett. 26, 3273–3276 (1999).

[b7] Hudson M. K., Elkington S. R., Lyon J. G. & Goodrich C. C. Increase in relativistic electron flux in the inner magnetosphere: ULF wave mode structure. Adv. Space Res. 25, 2327–2337 (2000).

[b8] Elkington S. R., Hudson M. K. & Chan A. A. Resonant acceleration and diffusion of outer zone electrons in an asymmetric geomagnetic field. J. Geophys. Res. 108, 1116 (2003).

[b9] Fei Y., Chan A. A., Elkington S. R. & Wiltberger M. J. Radial diffusion and MHD particle simulations of relativistic electron transport by ULF waves in the september 1998 storm. J. Geophys. Res. 111, A12209 (2006).

[b10] Mauk B. H. *et al.* Science objectives and rationale for the radiation belt storm probes mission. Space Sci. Rev. 179, 3–27 (2013).

[b11] Reeves G. D. *et al.* Electron acceleration in the heart of the Van Allen radiation belts. Science 341, 991–994 (2013). 23887876 10.1126/science.1237743

[b12] Thorne R. M. *et al.* Rapid local acceleration of relativistic radiation-belt electrons by magnetospheric chorus. Nature 504, 411–414 (2013). 24352287 10.1038/nature12889

[b13] Roederer J. G. Dynamics of Geomagnetically Trapped Radiation Springer-Verlag (1970).

[b14] Baker D. N. *et al.* Coronal mass ejections, magnetic clouds, and relativistic magnetospheric electron events: ISTP. J. Geophys. Res. 103, 17279–17292 (1998).

[b15] Rostoker G., Skone S. & Baker D. N. On the origin of relativistic electrons in the magnetosphere associated with some geomagnetic storms. Geophys. Res. Lett. 25, 3701–3704 (1998).

[b16] Mathie R. A. & Mann I. R. A correlation between extended intervals of ULF wave power and storm-time geosynchronous relativistic electron flux enhancements. Geophys. Res. Lett. 27, 3261–3264 (2000).

[b17] O'Brien T. P. *et al.* Energization of relativistic electrons in the presence of ULF power and MeV microbursts: Evidence for dual ULF and VLF acceleration. J. Geophys. Res. 108, 1329 (2003).

[b18] Ukhorskiy A. Y., Anderson B. J., Takahashi K. & Tsyganenko N. A. Impact of ULF oscillations in solar wind dynamic pressure on the outer radiation belt electrons. Geophys. Res. Lett. 33, L06111 (2006).

[b19] Reeves G. D. *et al.* The global response of relativistic radiation belt electrons to the January 1997 magnetic cloud. Geophys. Res. Lett. 25, 3265–3268 (1998).

[b20] Loto'Aniu T. M. *et al.* Radial diffusion of relativistic electrons into the radiation belt slot region during the 2003 Halloween geomagnetic storms. J. Geophys. Res. 111, 4218 (2006).

[b21] Horne R. B. *et al.* Wave acceleration of electrons in the Van Allen radiation belts. Nature 437, 227–230 (2005). 16148927 10.1038/nature03939

[b22] Shprits Y. Y. *et al.* Acceleration mechanism responsible for the formation of the new radiation belt during the 2003 halloween solar storm. Geophys. Res. Lett. 33, L05104 (2006).

[b23] Baker D. N. *et al.* The relativistic electron-proton telescope (REPT) instrument on board the radiation belt storm probes (RBSP) spacecraft: Characterization of Earth's radiation belt high-energy particle populations. Space Sci. Rev. 179, 337–381 (2013).

[b24] Blake J. B. *et al.* The magnetic electron ion spectrometer (MagEIS) instruments aboard the radiation belt storm probes (RBSP) spacecraft. Space Sci. Rev. 179, 383–421 (2013).

[b25] Spence H. E. *et al.* Science goals and overview of the energetic particle, composition, and thermal plasma (ECT) suite on NASAs Radiation Belt Storm Probes (RBSP) mission. Space Sci. Rev. 179, 311–336 (2013).

[b26] Wygant J. *et al.* The electric field and waves instruments on the radiation belt storm probes mission. Space Sci. Rev. 179, 183–220 (2013).

[b27] Kletzing C. A. *et al.* The electric and magnetic field instrument suite and integrated Science (EMFISIS) on RBSP. Space Sci. Rev. 179, 127–181 (2013). 10.1007/s11214-023-00973-z PMC10129970 37123883

[b28] McIlwain C. E. Coordinates for mapping the distribution of magnetically trapped particles. J. Geophys. Res. 66, 3681–3691 (1961).

[b29] Southwood D. J. & Kivelson M. G. Charged particle behavior in low-frequency geomagnetic pulsations. II - Graphical approach. J. Geophys. Res. 87, 1707–1710 (1982).

[b30] Zong Q. *et al.* Ultralow frequency modulation of energetic particles in the dayside magnetosphere. Geophys. Res. Lett. 34, L12105 (2007).

[b31] Claudepierre S. G. *et al.* Van Allen Probes observation of localized drift resonance between poloidal mode ultra-low frequency waves and 60 keV electrons. Geophys. Res. Lett. 40, 4491–4497 (2013).

[b32] Torrence C. & Compo G. P. A practical guide to wavelet analysis. Bull. Amer. Meteor. Soc. 79, 61–78 (1998).

[b33] Mann I. R. *et al.* Discovery of the action of a geophysical synchrotron in the Earth's Van Allen radiation belts. Nat. Commun. 4, 2795 (2013).

[b34] Tu W., Elkington S. R., Li X., Liu W. & Bonnell J. Quantifying radial diffusion coefficients of radiation belt electrons based on global MHD simulation and spacecraft measurements. J. Geophys. Res. 117, 10210 (2012).

[b35] Green J. C. & Kivelson M. G. Relativistic electrons in the outer radiation belt: differentiating between acceleration mechanisms. J. Geophys. Res. 109, A03213 (2004).

[b36] Chen Y., Reeves G. D. & Friedel R. H. W. The energization of relativistic electrons in the outer Van Allen radiation belt. Nat. Phys. 3, 614–617 (2007).

[b37] Su Z., Xiao F., Zheng H. & Wang S. STEERB: a three-dimensional code for storm-time evolution of electron radiation belt. J. Geophys. Res. 115, A09208 (2010).

[b38] Brautigam D. H. *et al.* CRRES electric field power spectra and radial diffusion coefficients. J. Geophys. Res. 110, A02214 (2005).

[b39] Huang C.-L., Spence H. E., Singer H. J. & Hughes W. J. Modeling radiation belt radial diffusion in ULF wave fields: 1. Quantifying ULF wave power at geosynchronous orbit in observations and in global MHD model. J. Geophys. Res. 115, A06215 (2010).

[b40] Ozeke L. G. *et al.* ULF wave derived radiation belt radial diffusion coefficients. J. Geophys. Res. 117, A04222 (2012). 10.1002/2013JA019204 PMC4497482 26167440

[b41] Rae I. J. *et al.* Ground-based magnetometer determination of in situ Pc4-5 ULF electric field wave spectra as a function of solar wind speed. J. Geophys. Res. 117, A04221 (2012).

[b42] Mann I. R. *et al.* Dynamics of the Earth's Radiation Belts and Inner Magnetosphere 199, 69–92 (2012).

[b43] Ali A. F. *et al.* Magnetic field power spectra and magnetic radial diffusion coefficients using CRRES magnetometer data. J. Geophys. Res. 120, 973–995 (2015).

[b44] Takahashi K. *et al.* Externally driven plasmaspheric ULF waves observed by the Van Allen Probes. J. Geophys. Res. 120, 526–552 (2015).

[b45] Meredith N. P., Horne R. B. & Anderson R. R. Substorm dependence of chorus amplitudes: Implications for the acceleration of electrons to relativistic energies. J. Geophys. Res. 106, 13165–13178 (2001).

[b46] Summers D. *et al.* Model of the energization of outer-zone electrons by whistler-mode chorus during the October 9, 1990 geomagnetic storm. Geophys. Res. Lett. 29, 2174 (2002).

[b47] Horne R. B. *et al.* Timescale for radiation belt electron acceleration by whistler mode chorus waves. J. Geophys. Res. 110, A03225 (2005). 10.1029/2019GL083071 PMC6772095 31598019

[b48] Thorne R. M. *et al.* Refilling of the slot region between the inner and outer electron radiation belts during geomagnetic storms. J. Geophys. Res. 112, A06203 (2007).

[b49] Li W. *et al.* Global distribution of whistler-mode chorus waves observed on the THEMIS spacecraft. Geophys. Res. Lett. 36, L09104 (2009).

[b50] Meredith N. P. *et al.* Global model of lower band and upper band chorus from multiple satellite observations. J. Geophys. Res. 117, 10225 (2012). 10.1002/2013GL059050 PMC4373175 25821274

[b51] Horne R. B. *et al.* A new diffusion matrix for whistler mode chorus waves. J. Geophys. Res. 118, 6302–6318 (2013).

[b52] Su Z. *et al.* Nonstorm time dynamics of electron radiation belts observed by the Van Allen Probes. Geophys. Res. Lett. 41, 229–235 (2014).

[b53] Su Z. *et al.* Intense duskside lower band chorus waves observed by Van Allen Probes: Generation and potential acceleration effect on radiation belt electrons. J. Geophys. Res. 119, 4266–4273 (2014).

[b54] Xiao F. *et al.* Wave-driven butterfly distribution of Van Allen belt relativistic electrons. Nat. Commun. 6, 8590 (2015). 26436770 10.1038/ncomms9590 PMC4600758

[b55] Albert J. M., Meredith N. P. & Horne R. B. Three-dimensional diffusion simulation of outer radiation belt electrons during the october 9, 1990, magnetic storm. J. Geophys. Res. 114, A09214 (2009).

[b56] Shprits Y. Y., Subbotin D. & Ni B. Evolution of electron fluxes in the outer radiation belt computed with the VERB code. J. Geophys. Res. 114, A11209 (2009).

[b57] Su Z., Xiao F., Zheng H. & Wang S. Radiation belt electron dynamics driven by adiabatic transport, radial diffusion, and wave-particle interactions. J. Geophys. Res. 116, A04205 (2011).

[b58] Takahashi K., McEntire R. W., Lui A. T. Y. & Potemra T. A. Ion flux oscillations associated with a radially polarized transverse Pc 5 magnetic pulsation. J. Geophys. Res. 95, 3717–3731 (1990).

[b59] Kurth W. S. *et al.* Electron densities inferred from plasma wave spectra obtained by the Waves instrument on Van Allen Probes. J. Geophys. Res. 120, 904–914 (2015). 10.1002/2014JA020857 PMC4497465 26167442

[b60] Tsyganenko N. A. & Sitnov M. I. Modeling the dynamics of the inner magnetosphere during strong geomagnetic storms. J. Geophys. Res. 110, A03208 (2005).

