# Supplementary material for: Ultra-low-frequency wave-driven diffusion of radiation belt relativistic electrons
Source: Nat Commun. 2015 Dec 22;6:10096. doi: 10.1038/ncomms10096 (PMC4703845; doi:10.1038/ncomms10096)
Supplement: Supplementary Information — Supplementary Figures 1-14, Supplementary Tables 1-3, Supplementary Notes 1-3 and Supplementary References [file ncomms10096-s1.pdf]

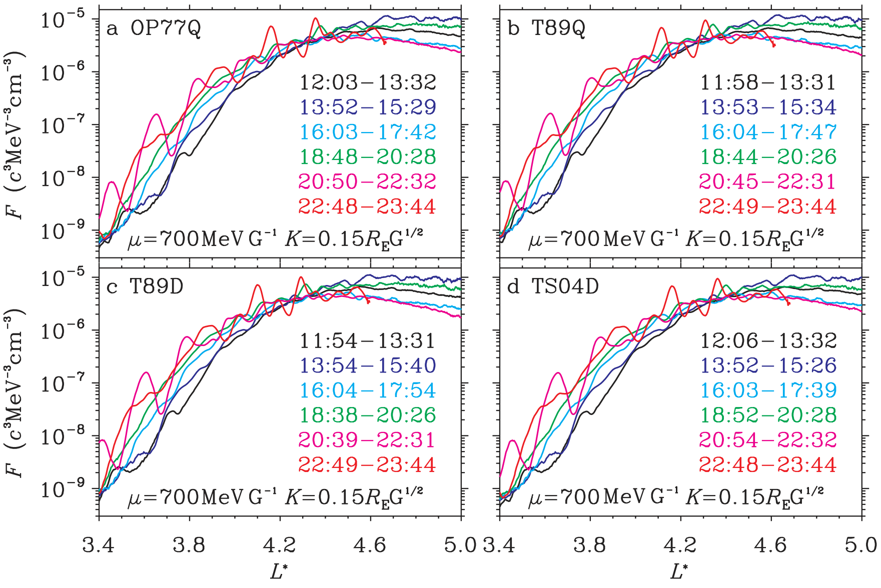

Supplementary Figure 1 | Radial profiles of relativistic electron phase space densities in the different magnetic field models for the 15 February 2014 event.

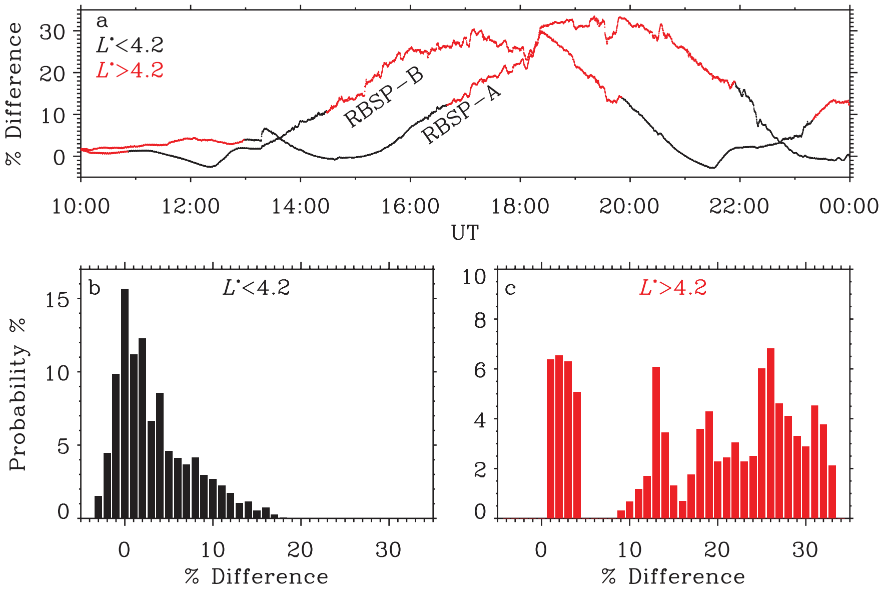

Supplementary Figure 2 | Relative difference between observed and TS04D-modeled geomagnetic fields for the 15 February 2014 event. Relative difference is defined as $(B_{\mathrm{O}} - B_{\mathrm{M}})/B_{\mathrm{O}}$ with the observed B_{O} and modeled B_{M} magnetic fields. (**a**) Spatiotemporal-dependence of the relative difference. (**b,c**) Probability distributions of the relative difference in the regions $L^* < 4.2$ and $L^* > 4.2$.

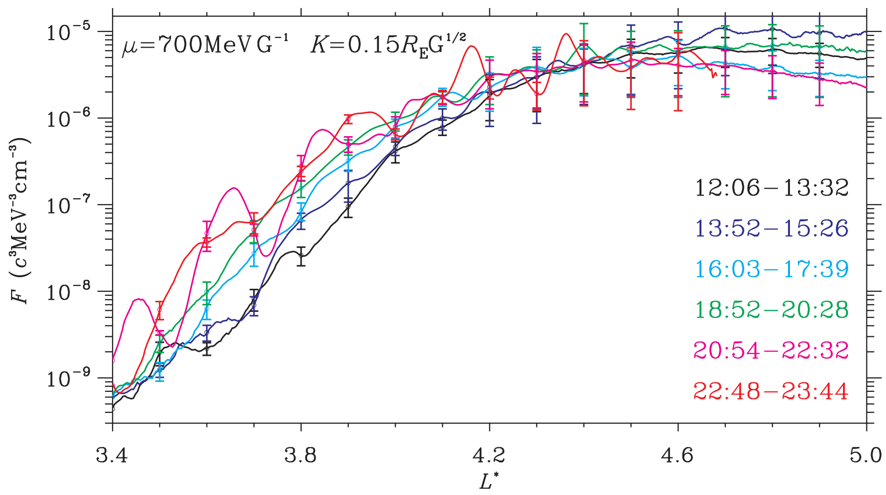

Supplementary Figure 3 | Radial profiles of relativistic electron phase space densities in the TS04D geomagnetic field model for the 15 February 2014 event. The error bars associated with the average inaccuracy of magnetic field model are overplotted every 0.1 L^*-shell (calculation details are given in Supplementary Note 1).

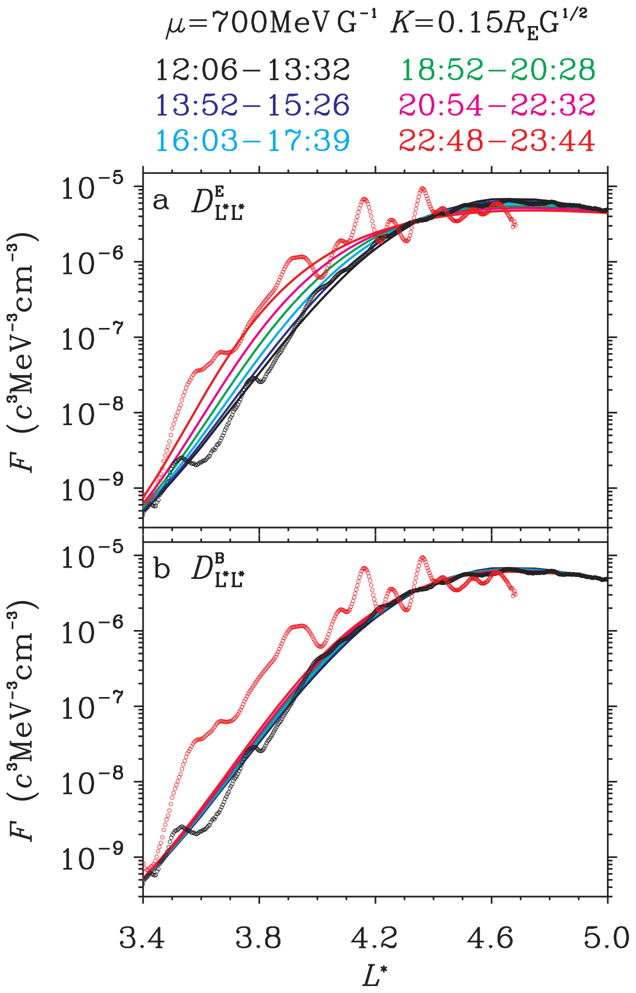

Supplementary Figure 4 | Simulated electron phase space density evolution driven only by (a) electric, or (b) magnetic perturbations of ULF waves.

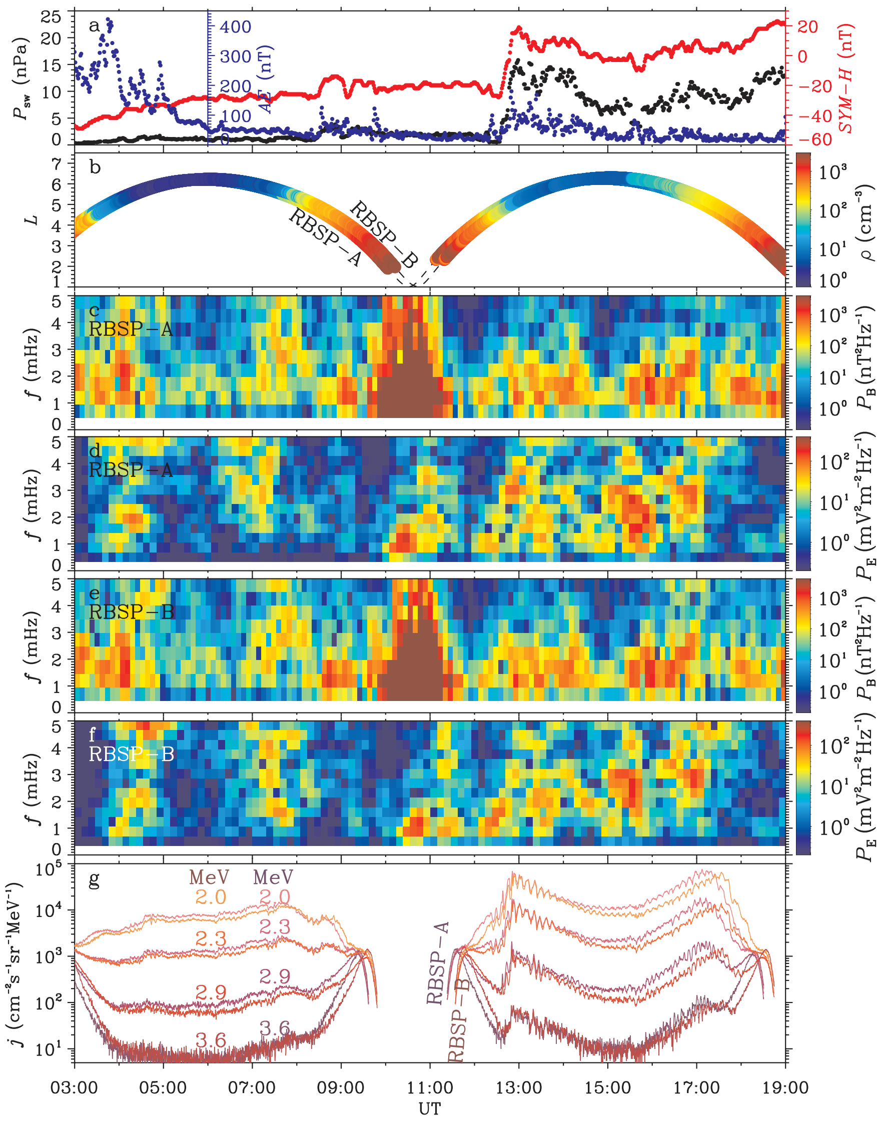

Supplementary Figure 5 | An overview of the 18 January 2013 radiation belt event. (**a**) Solar wind dynamic pressure P_{sw}, geomagnetic activity indices *AE* and *SYM-H*. (**b**) Cold electron number density ρ from the EMFISIS suite[1]. (**c,e**) Power spectral density P_{B} of the compressional ULF wave magnetic field in the MFA coordinate system from the EMFISIS magnetometer. (**d,f**) Power spectral density P_{E} of the y-component ULF wave electric field in the mGSE coordinate system from the EFW instrument. (**g**) Spin-averaged differential electron fluxes j (color-coded according to energy) in the outer radiation belt from the REPT instrument.

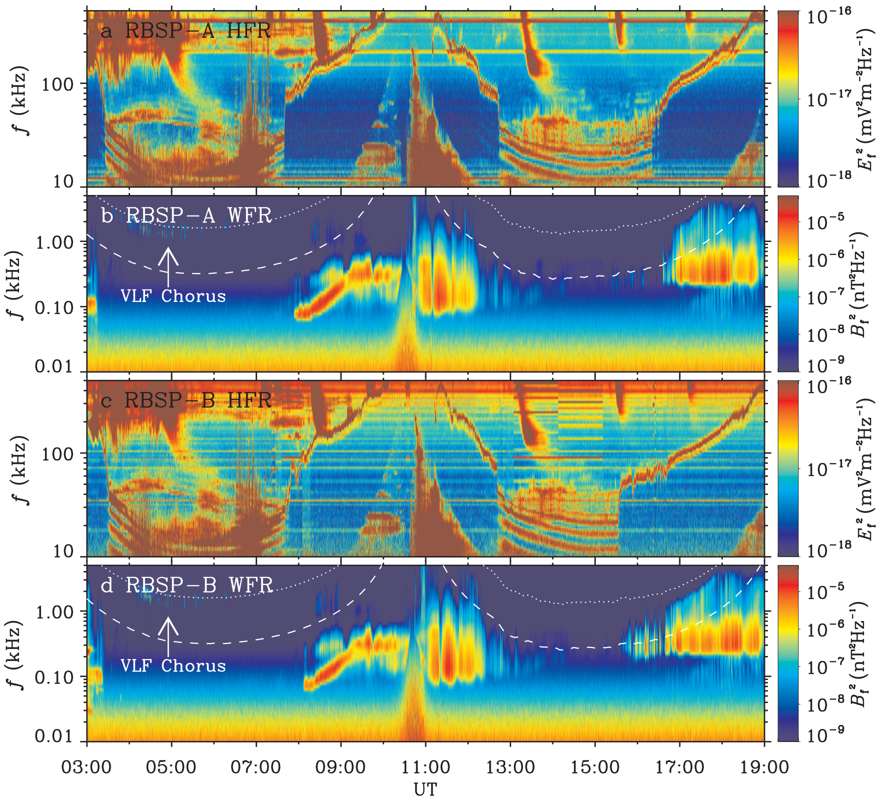

Supplementary Figure 6 | Electromagnetic power spectral densities of high frequency and very low frequency waves for the 18 January 2013 event. (**a,c**) Wave electric power spectral density from the EMFISIS-HFR instrument. The steep variation in the upper hybrid band indicated the plasmapause crossing. (**b,d**) Wave magnetic power spectral density from the EMFISIS-WFR instrument. The dashed and dotted lines represent 0.1 f_{ce} and 0.5 f_{ce} with the equatorial electron gyrofrequency f_{ce} determined based on the TS04D geomagnetic model.

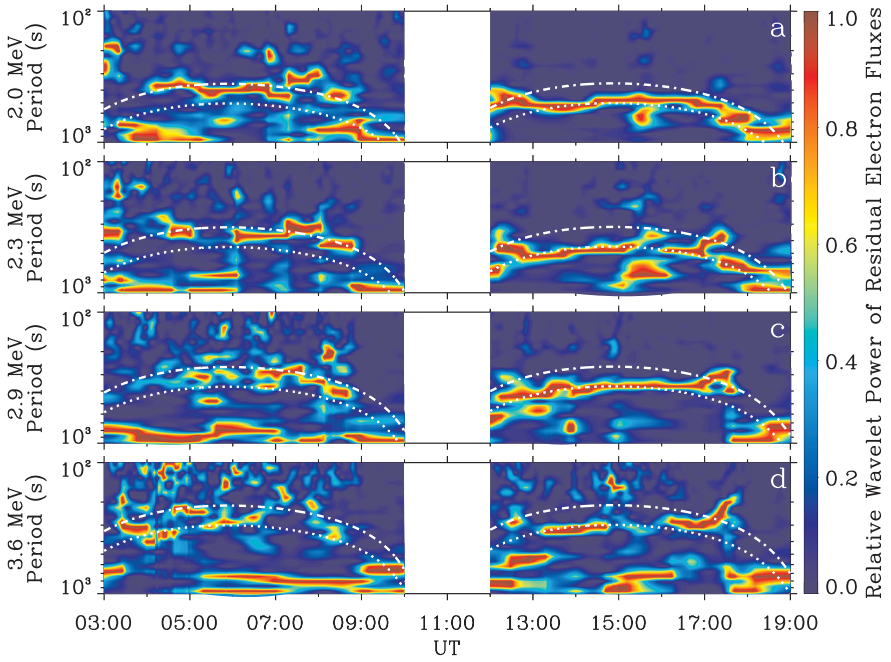

Supplementary Figure 7 | Relative wavelet power of residual electron fluxes observed by Van Allen Probe A for the 18 January 2013 event. Residual flux is defined as $(\bar{j} - j_0)/j_0$, slightly different from that of Fig. 3 (in main text). Here $\bar{j}$ and j_0 represent the 100 s and 1000 s running averaged j with the spin-averaged differential flux j from the REPT instrument. The additional 100 s running average process is performed to reduce the high-frequency noise particularly at the energy channel 3.6 MeV (Supplementary Fig. 5g). The superposed dotted and dot-dashed lines represent the drift periods of electrons with the equatorial pitch-angles $0°$ and $90°$ at the corresponding energy channels in a dipole field. Note that Van Allen Probe B gave the results generally consistent with those from Van Allen Probe A.

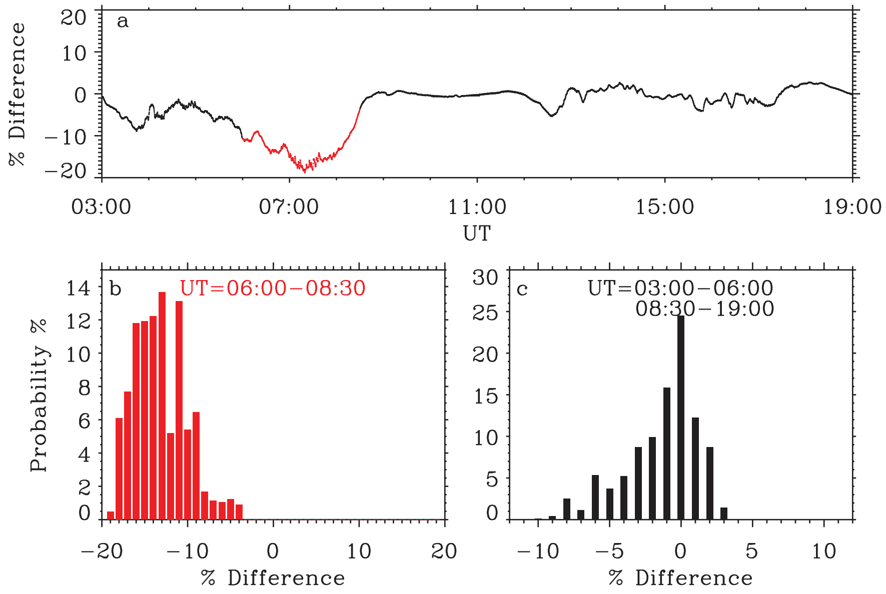

Supplementary Figure 8 | Relative difference between Van Allen Probe A observed and TS04D modeled geomagnetic fields for the 18 January 2013 event. Relative difference is defined as $(B_{\rm O} - B_{\rm M})/B_{\rm O}$ with the observed $B_{\rm O}$ and modeled $B_{\rm M}$ magnetic fields. (**a**) Temporal-dependence of the relative difference. (**b,c**) Probability distributions of the relative difference in the different time ranges. Note that Van Allen Probe B gave the results generally consistent with those from Van Allen Probe A.

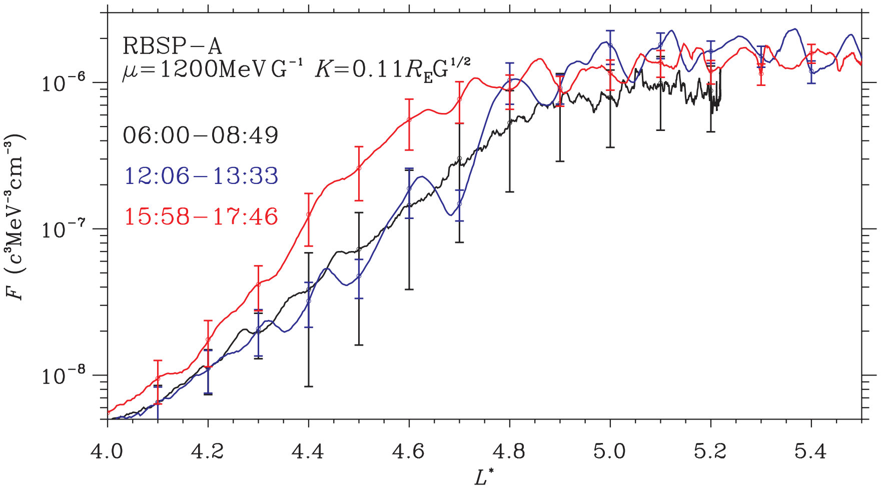

Supplementary Figure 9 | Radial profiles of Van Allen Probe A observed relativistic electron phase space densities in the TS04D geomagnetic field model for the 18 January 2013 event. The error bars associated with the average inaccuracy of magnetic field model are overplotted every 0.1 L^*-shell (calculation details are given in Supplementary Note 1). Note that Van Allen Probe B gave the results generally consistent with those from Van Allen Probe A.

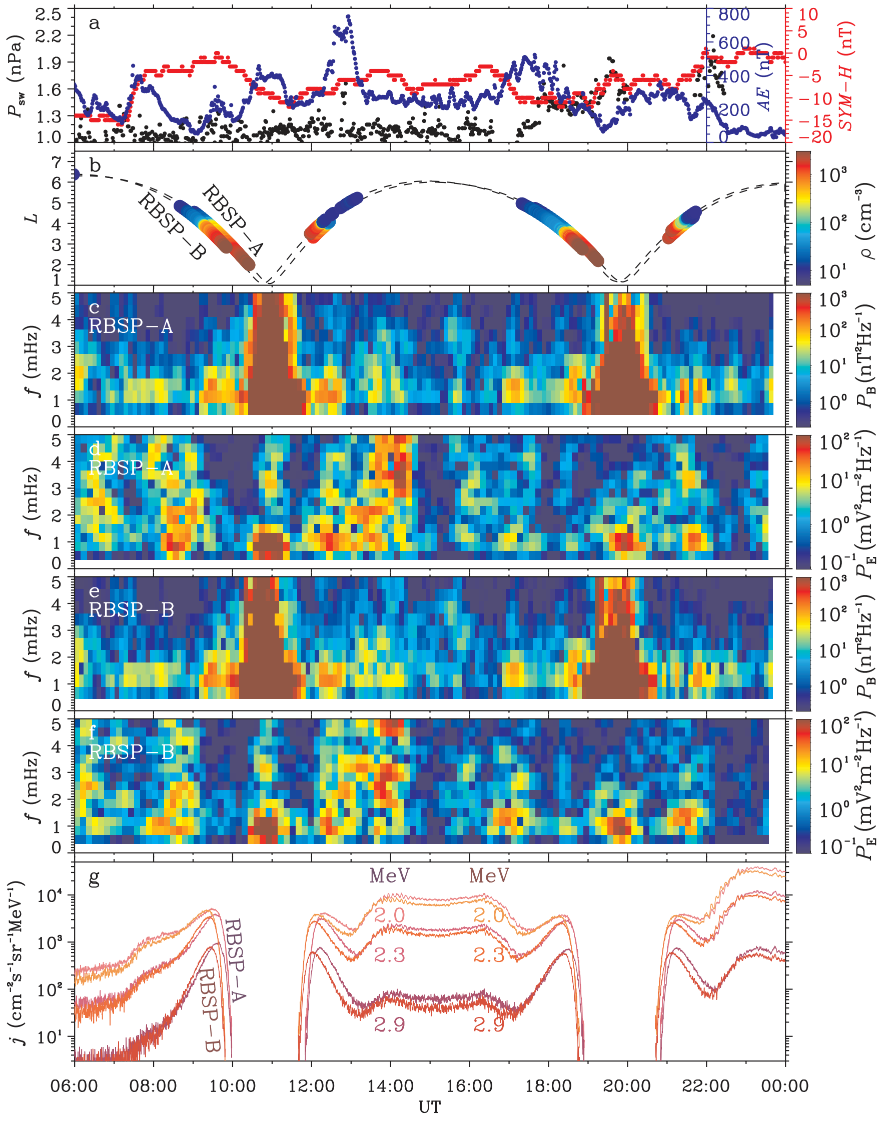

Supplementary Figure 10 | An overview of the 22 September 2014 radiation belt event. (**a**) Solar wind dynamic pressure P_{sw}, geomagnetic activity indices *AE* and *SYM-H*. (**b**) Cold electron number density ρ from the EFW instrument. (**c,e**) Power spectral density P_{B} of the compressional ULF wave magnetic field in the MFA coordinate system from the EMFISIS magnetometer. (**d,f**) Power spectral density P_{E} of the y-component ULF wave electric field in the mGSE coordinate system from the EFW instrument. (**g**) Spin-averaged differential electron fluxes j (color-coded according to energy) in the outer radiation belt from the REPT instrument.

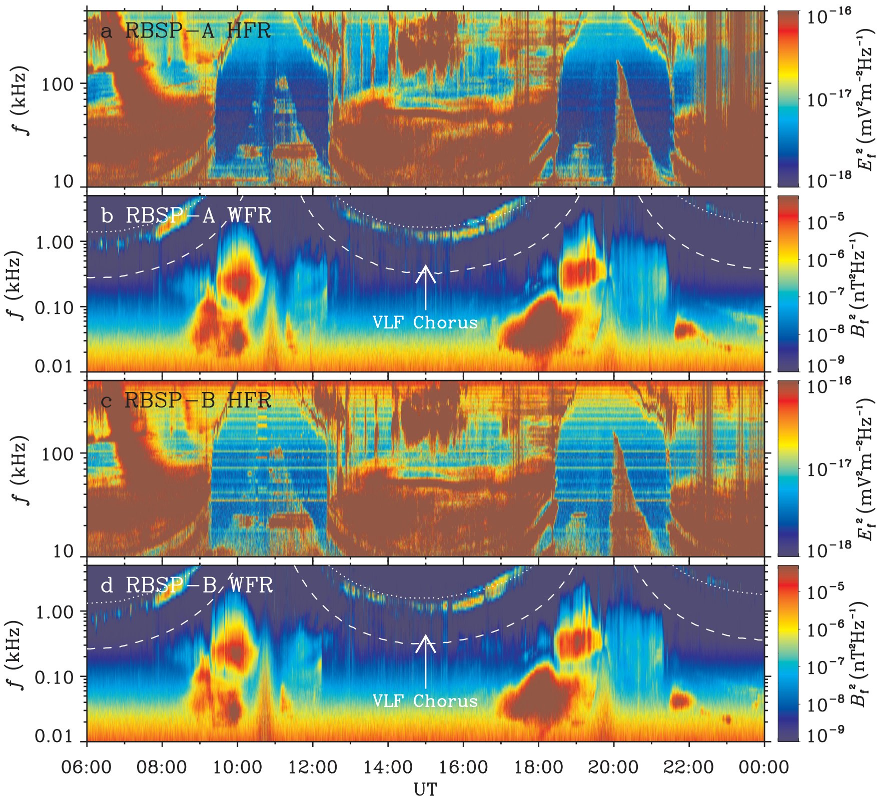

Supplementary Figure 11 | Electromagnetic power spectral densities of high frequency and very low frequency waves for the 22 September 2014 event. (**a,c**) Wave electric power spectral density from the EMFISIS-HFR instrument. The steep variation in the upper hybrid band indicated the plasmapause crossing. (**b,d**) Wave magnetic power spectral density from the EMFISIS-WFR instrument. The dashed and dotted lines represent 0.1 f_{ce} and 0.5 f_{ce} with the equatorial electron gyrofrequency f_{ce} determined based on the TS04D geomagnetic model.

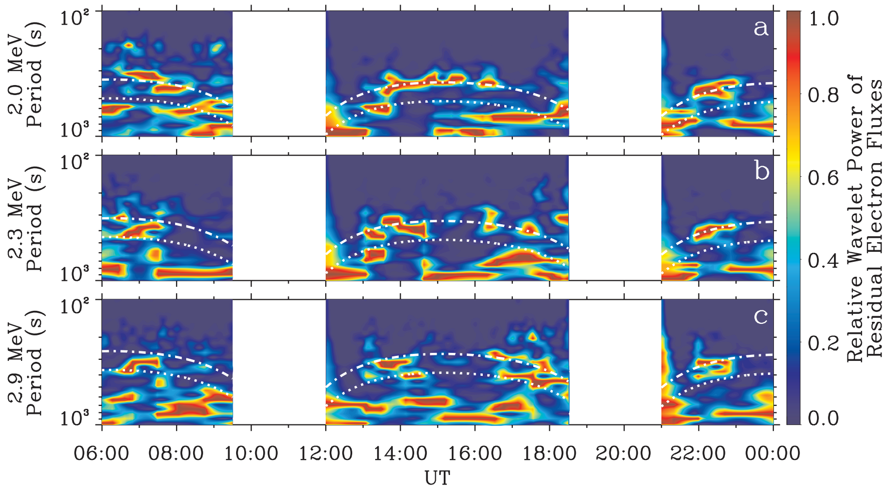

Supplementary Figure 12 | Relative wavelet power of residual electron fluxes observed by Van Allen Probe A for the 22 September 2014 event. Here the definition of residual flux is the same as that in Supplementary Fig. 7. The superposed dotted and dot-dashed lines represent the drift periods of electrons with the equatorial pitch-angles $0°$ and $90°$ at the corresponding energy channels in a dipole field. In the spatial region $L \approx 4.0$–6.0 (around 14:00 UT, 16:30 UT and 22:30 UT), the dominant oscillation periods were close to the corresponding electron drift periods. Note that Van Allen Probe B gave the results generally consistent with those from Van Allen Probe A.

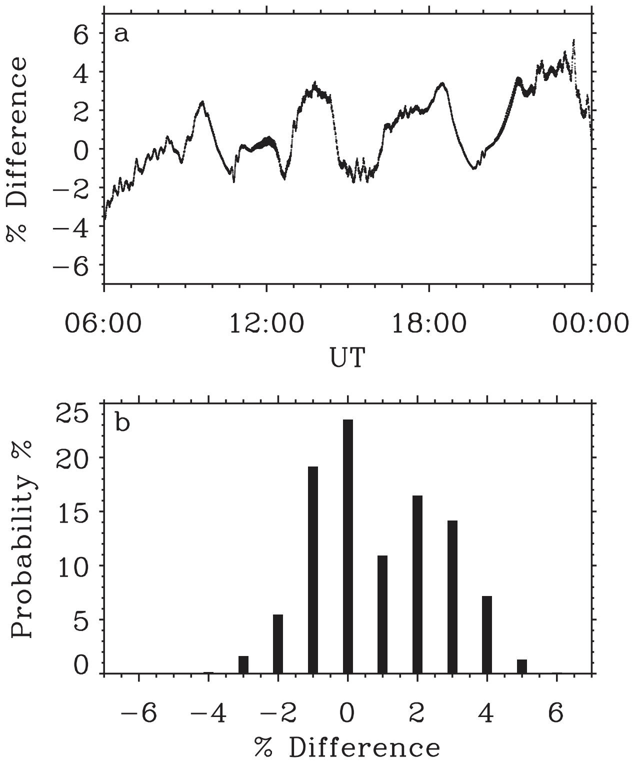

Supplementary Figure 13 | Relative difference between Van Allen Probe A observed and TS04D modeled geomagnetic fields for the 22 September 2014 event. Relative difference is defined as $(B_\mathrm{O} - B_\mathrm{M})/B_\mathrm{O}$ with the observed B_O and modeled B_M magnetic fields. (**a**) Temporal-dependence of the relative difference. (**b**) Probability distributions of the relative difference throughout the event. Note that Van Allen Probe B gave the results generally consistent with those from Van Allen Probe A.

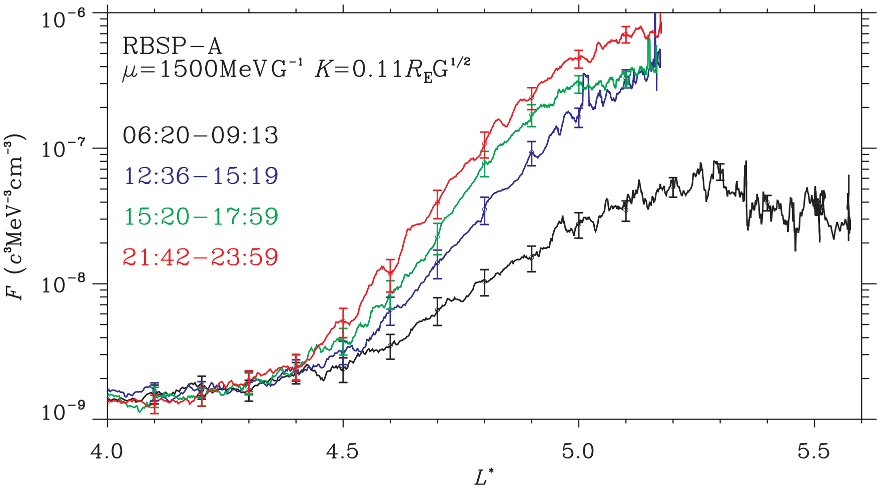

Supplementary Figure 14 | Radial profiles of Van Allen Probe A observed relativistic electron phase space densities in the TS04D geomagnetic field model for the 22 September 2014 event. The error bars associated with the average inaccuracy of magnetic field model are overplotted every 0.1 L^*-shell (calculation details are given in Supplementary Note 1). Note that Van Allen Probe B gave the results generally consistent with those from Van Allen Probe A.

Supplementary Table 1 | Spatial locations of Van Allen Probes at the selected times for the 15 February 2014 event.

UT	10:00	12:00	14:00	16:00	18:00	20:00	22:00	00:00
				Van Allen Probe A				
MLT (h)	12.53	13.89	16.76	9.19	11.32	12.61	14.34	3.30
MLAT (deg)	−12.18	−8.48	−3.81	−1.26	−0.72	0.43	3.85	−7.44
L	6.03	5.38	2.59	3.41	5.49	5.68	4.29	1.26
				Van Allen Probe B				
MLT (h)	14.03	17.46	9.49	11.43	12.67	14.42	4.74	10.28
MLAT (deg)	−13.42	−7.51	−0.25	−0.27	1.41	5.03	−13.45	−16.11
L	5.36	2.19	3.72	5.60	5.70	4.20	1.54	5.19

Supplementary Table 2 | Spatial locations of Van Allen Probes at the selected times for the 18 January 2013 event.

UT	03:00	05:00	07:00	09:00	11:00	13:00	15:00	17:00	19:00
					Van Allen Probe A				
MLT (h)	23.86	2.32	4.06	6.19	21.70	1.81	3.08	4.34	8.37
MLAT (deg)	−1.65	−0.70	2.10	6.44	−14.42	−11.50	−10.92	−9.18	6.65
L	3.96	6.01	5.93	4.00	1.98	5.36	6.25	5.17	1.88
					Van Allen Probe B				
MLT (h)	23.59	2.17	3.91	5.89	20.06	1.58	2.90	4.11	7.15
MLAT (deg)	−1.21	−0.69	1.91	5.92	−13.48	−12.22	−11.67	−10.32	0.74
L	3.73	5.96	6.03	4.28	1.49	5.19	6.31	5.43	2.37

Supplementary Table 3 | Spatial locations of Van Allen Probes at the selected times for the 22 September 2014 event.

UT	06:00	08:00	10:00	12:00	14:00	16:00	18:00	20:00	22:00	00:00
					Van Allen Probe A					
MLT (h)	4.84	6.34	9.04	1.39	3.73	5.05	6.76	18.67	2.48	4.19
MLAT (deg)	13.67	11.61	2.58	9.55	9.62	5.70	0.77	2.50	1.61	0.03
L	6.29	5.66	2.86	3.24	5.71	5.85	4.33	1.24	4.73	5.90
					Van Allen Probe B					
MLT (h)	4.79	6.31	9.42	1.54	3.65	4.95	6.73	20.15	2.41	4.07
MLAT (deg)	14.22	12.21	1.19	9.97	9.79	6.07	1.31	2.63	1.94	0.82
L	6.38	5.56	2.50	3.57	5.84	5.84	4.17	1.42	4.89	5.96

Supplementary Note 1 | Uncertainty of electron phase space density

The electron phase space density (PSD) is a function of the adiabatic invariants whose calculation requires information about the global configuration of the geomagnetic field[2,3]. Such global configuration cannot be obtained from the local satellite measurements and has to be described by an appropriate magnetic field model. An assumed geomagnetic field model can lead to the generation of errors on the PSD profiles[2,3]. We have analyzed the radial profiles of relativistic electron PSDs in the four different magnetic field models (Supplementary Fig. 1): OP77Q[4], T89Q[3,5], T89D[5] and TS04D[6]. These models give a qualitative consistent picture, illustrating the earthward movement of the inner edge of the outer radiation belt. The difference between observed and TS04D-modeled geomagnetic fields (Supplementary Fig. 2) was relatively small (averagely 4%) in the region $L^* < 4.2$, but became much larger (averagely 18%) in the region $L^* > 4.2$. Such large differences were probably produced by the enhanced compression of dayside magnetosphere (Fig. 1a in main text). The uncertainties of the magnetic field amplitude translate to the uncertainties in the kinetic energy for a fixed first adiabatic invariant, and then the corresponding uncertainties in the electron flux and PSD can be calculated based on the observed energy spectrum[3]. The errors associated with the average inaccuracy of TS04D geomagnetic field model are found to be smaller than the extent of electron PSD variation in the slot region (Supplementary Fig. 3).

Supplementary Note 2 | Relative importance of electric and magnetic perturbations

To illustrate the relative importance of electric and magnetic perturbations of ULF waves, we have performed the radial diffusion simulations driven by $D^{\mathrm{E}}_{\mathrm{L^*L^*}}$ or $D^{\mathrm{B}}_{\mathrm{L^*L^*}}$ alone (Supplementary Fig. 4). The electric perturbations were able to largely explain the electron PSD evolution, while the contribution of magnetic perturbations was quite limited.

Supplementary Note 3 | Radiation belt events under different magnetospheric conditions

By analyzing a radiation belt event in the plasmasphere without detectable VLF chorus waves, we have illustrated the ability of ULF waves to radially diffuse the relativistic electrons. In fact, the radial diffusion characteristics can still be found under other different magnetospheric conditions. Here we show two more radiation belt events with the concurrence of ULF and VLF chorus waves.

Event on 18 January 2013 This radiation belt event occurred during the recovery phase of a moderate geomagnetic storm (Supplementary Fig. 5a) with the plasmapause at L =4.5–5.0 (Supplementary Figs 5b, 6a and 6c and Supplementary Table 2). Weak VLF chorus waves (with the power spectral densities below 10^{-7} $\mathrm{nT^2Hz^{-1}}$) were detected in the nightside region (MLT=0–6) outside the plasmapause before 10:00 UT (Supplementary Figs 6b and 6d and Supplementary Table 2). In contrast, the ULF waves (Supplementary Figs 5c–5f) can be observed clearly throughout the event. Particularly after 12:30 UT, the sudden increase in the solar wind dynamic pressure excited strong ULF waves and then induced the significant oscillations of relativistic electron fluxes (Supplementary Fig. 5g). The flux oscillation periods were close to the corresponding electron drift periods

(Supplementary Fig. 7), suggesting the occurrence of $m = 1$ mode drift-resonance between ULF waves and relativistic electrons. We adopt the TS04D geomagnetic field model (Supplementary Fig. 8) to calculate the adiabatic invariants and the relativistic electron PSDs (Supplementary Fig. 9). The relative difference between the observed and modeled magnetic fields were large (averagely 15%) during the time range 06:00-8:30 UT, and became quite small (averagely 4%) in the remaining time range. The obtained relativistic electron PSDs appeared to be monotonic throughout the event, suggesting the dominance of radial diffusion over local acceleration. The inner edge of outer belt moved earthward about 0.25 R_{E} during 10 hours, mainly produced by the ULF-wave driven radial diffusion.

Event on 22 September 2014 This radiation belt event occurred under the non-storm condition (Supplementary Fig. 10a) with the plasmapause at L =4.0 (Supplementary Figs 10b, 11a and 11c and Supplementary Table 3). Strong VLF chorus waves (with the power spectral densities up to 10^{-5} $\mathrm{nT^2Hz^{-1}}$) were detected in the nightside region (MLT=0–6) outside the plasmapause (Supplementary Figs 11b and 11d and Supplementary Table 3), significantly different from the events on 18 January 2013 and 15 February 2014. The observed ULF waves (Supplementary Figs 10c–10f) were weaker than those in the 18 January 2013 and February 15 2014 events, but still caused the obvious oscillations of relativistic electron fluxes (Supplementary Fig. 10g) in the spatial region $L \approx 4.0$–6.0 (around 14:00 UT, 16:30 UT and 22:30 UT). The occurrence of $m = 1$ mode drift-resonance driven by ULF waves was supported by the wavelet transform analysis for the electron fluxes (Supplementary Fig. 12). The TS04D geomagnetic field model well reproduced the observed magnetic fields (with the relative difference 2% on average) throughout the event

(Supplementary Fig. 13), and the obtained relativistic electron PSDs behaved quite differently from those of the events on 18 January 2013 and 15 February 2014 (Supplementary Fig. 14). The local acceleration by VLF chorus waves explained the formation of a significant peak in the relativistic PSD around $L^* = 5.2$. In the region $L^* < 5.2$ with steep gradients of PSDs, the radial diffusion by ULF waves could redistribute the electrons toward the Earth.

Supplementary References

1. Kurth, W. S. *et al.* Electron densities inferred from plasma wave spectra obtained by the Waves instrument on Van Allen Probes. *J. Geophys. Res.* **120**, 904–914 (2015).

2. Green, J. C. & Kivelson, M. G. Relativistic electrons in the outer radiation belt: Differentiating between acceleration mechanisms. *J. Geophys. Res.* **109**, A03213 (2004).

3. Reeves, G. D. *et al.* Electron acceleration in the heart of the Van Allen radiation belts. *Science* **341**, 991–994 (2013).

4. Olson, W. P. & Pfitzer, K. A. A dynamic model of the magnetospheric magnetic and electric fields for July 29, 1977. *J. Geophys. Res.* **87**, 5943–5948 (1982).

5. Tsyganenko, N. A. A magnetospheric magnetic field model with a warped tail current sheet. *Planet. Space Sci.* **37**, 5–20 (1989).

6. Tsyganenko, N. A. & Sitnov, M. I. Modeling the dynamics of the inner magnetosphere during strong geomagnetic storms. *J. Geophys. Res.* **110**, A03208 (2005).
